# Cylindrical IR-ATR Sensors for Process Analytics

**DOI:** 10.3390/s20102917

**Published:** 2020-05-21

**Authors:** Armin Lambrecht, Carsten Bolwien, Jochen Erb, Hendrik Fuhr, Gerd Sulz

**Affiliations:** Fraunhofer IPM, Heidenhofstr. 8, D-79110 Freiburg, Germany; carsten.bolwien@ipm.fraunhofer.de (C.B.); jochen.erb@ipm.fraunhofer.de (J.E.); hendrik.fuhr@ipm.fraunhofer.de (H.F.); gerd.sulz@ipm.fraunhofer.de (G.S.)

**Keywords:** infrared spectroscopy, ATR (attenuated total reflection), PAT (process analytical technology), inline, online, isocyanate, acetonitrile, sapphire, photometer, miniature spectrometer

## Abstract

Infrared attenuated total reflection (ATR) spectroscopy is a common laboratory technique for the analysis of highly absorbing liquids and solids. However, in a process environment, maintaining a sufficient sample exchange and cleaning of the sensitive surface of the element is a crucial issue. An important industrial application is the measurement of isocyanate concentrations. Isocyanates are necessary for the fabrication of polyurethane materials and are among the chemicals with the highest production volume worldwide. For process applications, narrowband photometers or MEMS spectrometers are more appropriate than the use of bulky FTIR instruments frequently encountered in a laboratory environment. Toluene diisocyanate (TDI) and hexamethylene diisocyanate (HDI) concentrations are measured with a planar ATR photometer setup. Using a miniature Fabry–Perot interferometer (FPI), trace concentrations below 100 ppm (m/m) are detected. By employing an ATR element of the cylindrical shape, sensors can be realized with a smooth surface ideally suited for an automatic cleaning system in a process environment. A laboratory setup with sapphire tubes as ATR elements for incorporation in a liquid flow system is described. Reflection and transmission configurations were investigated. Measurements with acetonitrile as a less toxic substitute showed that with cylindrical ATR sensors’ detection limits for isocyanate concentrations below 100 ppm (m/m) are feasible.

## 1. Introduction

Infrared attenuated total reflection (ATR) spectroscopy is a common technique for the analysis of highly absorbing liquids and solids. When a radiation wave in an optical material is totally reflected at an interface with a material of lower index of refraction, part of the wave penetrates into the sample. The penetration depth of this evanescent wave, i.e., the probed sample thickness, is approximately equal to the radiation wavelength λ_s_ in the sample [1,2]. In the mid-infrared (MIR) range (3 < λ < 20µm), this is only a few µm. The ATR technique has found widespread applications, e.g., in medical diagnostics [3,4], food quality analysis [5], beverage industry [6,7], water contamination monitoring [8,9], determination of moisture in the transformer and lubrication oil [10], pharmaceutical process analytics [11], and monitoring of rubber polymerization [12].

However, usually, ATR spectroscopy is mainly used in a laboratory environment. ATR attachments are available for most Fourier transform infrared (FTIR) spectrometers, e.g., [13]. In this case, samples are often manually placed on top of or, in the case of solid samples, pressed onto a suitable ATR crystal surface. Immersion ATR probes for the spectroscopy of liquids, e.g., in a laboratory reactor, are also available [14,15,16]. Usually, they consist of infrared light guides or fibers, which are connected to an ATR prism at the tip of the probe.

Surface contamination or residuals on the ATR element will cause erroneous results. Thus, careful cleaning and/or rinsing of the surface between different samples is necessary. If ATR sensors are used with flowing media, sufficient exchange at the surface has to be ensured. If such a device is applied in an industrial online or inline configuration, it is placed close to a product-carrying tube or vessel in a production environment, where manual cleaning is hardly possible. This is, e.g., the case for the beverage industry, where process sensors have to cope with established cleaning in place (CIP) systems [6,7]. For typical prism tip probes, automatic cleaning is not easy [17]. Even with a flat ATR crystal, optimum packaging of the element is crucial to avoid sharp edges and undercuts where sample flow may be constrained, and fouling may occur. Wiping and liquid cleaning would be easier for smooth cylindrical ATR surfaces.

Additionally, a process sensor has to be very robust and comply with all the required safety regulations. Therefore, narrowband photometers or miniature MEMS spectrometers are more appropriate than the use of FTIR instruments generally encountered in a laboratory environment. They are also a key component for small portable ATR sensors [18].

An important industrial application is the measurement of isocyanate concentrations. Isocyanates are necessary for the fabrication of polyurethane (PU) materials, and are among the chemicals with the highest production volumes worldwide. PU materials are utilized for coatings, construction foams, mattresses, shoes, automotive components, and many other products. Laboratory measurements of isocyanate reactions using fiberoptic probes are reported in [19,20]. The infrared spectra of isocyanates are dominated by a characteristic absorption of the NCO-stretching vibration around 2270 cm^−1^. Fortunately, in this spectral region, features of other components in a mixture, e.g., solvents, are hardly observed. Thus, for the determination of isocyanate concentrations, the measurement with a two-channel photometric detector can be sufficient. By the selection of suitable filters, one detection channel is positioned at the NCO band, and the other channel serves as a reference placed at a spectral position with no isocyanate absorption. However, as the shape and position of individual isocyanates differ, and the change of solvents and other components may introduce a varying background, spectral measurements with a sufficient bandwidth and resolution are preferred. A microspectrometer based on a Fabry–Perot interferometer (FPI) can fulfil this task.

In this work, the BASF product Basonat HI100 (trimerized hexamethylene diisocyanate (HDI), with propylene carbonate and isopropanol as solvents was used. In addition, some experiments were performed with toluene 2,4-diisocyanate (TDI) in monochlorobenzene (MCB). Both isocyanates were used for experiments with planar ATR elements. They are highly toxic chemicals, e.g., the OSHA personal exposure limit of TDI is 5 ppb [21]. Therefore, we were looking for a nontoxic alternative for the spectroscopic test of different ATR setups in the laboratory. Acetonitrile (AC) was identified as a suitable material with good solubility in isopropanol and/or water. Thus, experiments with cylindrical ATR elements were performed with acetonitrile in isopropanol.

This paper is organized as follows. Starting with planar ATR configurations, Section 2 describes an ATR photometer setup and the corresponding measurements with isocyanate solutions, followed by a modified setup with an MEMS microspectrometer and experiments with Basonat in propylene carbonate. Finally, with a similar planar configuration coupled to an FTIR instrument, the ATR spectra of CO_2_ gas, isocyanate, and acetonitrile solutions were obtained. These provide a reference for the analysis of the following results on cylindrical ATR sensors. Section 3 introduces the concept and realization of ATR setups with sapphire tubes as ATR elements. In Section 4, the experimental results with those setups are presented, followed by a discussion of the obtained data in Section 5 and a comparison to the results on planar ATR configurations. Section 6 concludes and gives an outlook on future developments.

## 2. Planar ATR Sensors

### 2.1. Results on Isocyanate Solutions Using a Planar ATR Photometer

The use of an ATR photometer for isocyanate concentration determination was previously demonstrated in a non-English publication [22]. As our work is based on these earlier results, the setup is briefly described here.

The sensor consists of a thermal infrared emitter (micro light bulb from MGG, Wentorf, Germany in [22], later setup: IR-emitter JSIR350-5-BL-R-D3.6-2-A4, Micro-Hybrid Electronic GmbH, Hermsdorf, Germany), a sapphire ATR crystal, and a dual-band pyroelectric detector to register radiation in a spectral window around the main absorption band of the analyte (sample channel) and a reference channel in a spectral window with negligible sample absorption. For optimum light throughput in the crystal, additional coupling optics is employed. The pyrodetector in [22] is an LMM-242 detector from InfraTec GmbH, Dresden, Germany with two spectral filters integrated in a TO8-mount (Table 1). The sapphire element is 32.5 mm long and 10 mm wide. A thickness of 1 mm yields 9 reflections in the mid infrared (MIR) range on the sample side of the crystal. The result of a typical measurement is the ratio of the two detector voltages V_signal_/V_reference_. The signal acquisition electronics converts these voltages to counts/s, which are e.g. displayed in Section 4 (Figures 7–10). As the radiation source is operated with a low frequency modulation below 4 Hz, a narrow band lock-in detection scheme is employed.

Throughout this paper, dealing with spectroscopic and photometric data, we use absorbances calculated as the negative base-10-logarithm of the ratio of sample signals and reference signals. These signals may be measurements at a specific wavelength in spectroscopic data or integrated signals from photometric setups. For photometers, positive or negative absorbance values may occur, depending on, e.g., the employed filters. The resulting absorbances will be marked with the dimensionless unit AU or milli-AU (mAU) for absorbance units. Absorbance changes ΔA, e.g., between the absorbance of a pure solvent and of a sample solution, should be approximately proportional to the concentration.

**Figure 1:**
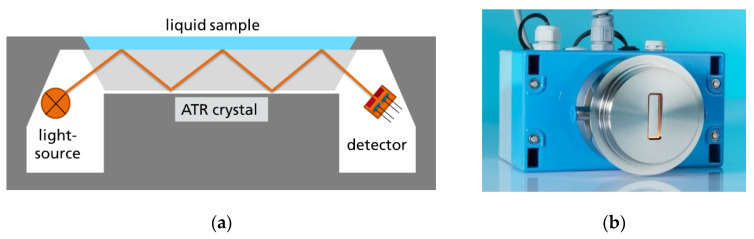
(**a**) Concept of a liquid sensor using a planar ATR element [22] and (**b**) picture of a later industrial sensor system for CO_2_ measurements in beverag.es.

Originally, the setup was designed to measure the CO_2_ content in beverages (Figure 1) [7], and a narrow bandpass filter (#1 s. Table 1) was employed for the signal channel. However, this filter only covers a small part of the isocyanate band, resulting in low signal voltages. With a broader bandpass (#2 s. Table 1), the isocyanate band was completely covered. A Varivent DN60 flange, common in beverage industry installations, was employed. The crystal was sealed with Teflon into the flange, and by a force-locked support it was integrated into a pressure-resistant enclosure.

In [22], a corresponding sensor setup was used for laboratory measurements at BASF. A cap on the sensor flange served as a flow cell, and solvents spiked with varying isocyanate concentrations were continuously pumped through this cell. As a result, with this photometric setup, the HDI and TDI concentrations in propylene carbonate respectively in MCB could be measured over the full concentration range from 0% to 100%.

For HDI, the BASF product Basonat HI 100 in propylene carbonate was used. For a 4600 ppm (m/m) solution, an absorbance change ΔA of 7.05 mAU is determined from Figure 8 in [22]. The standard deviation σ for an average time of 1 min is approximately 0.14 mAU. A noise equivalent concentration (NEC) (1σ) ≈ 91 ppm (m/m) indicates that a limit of detection below 200 ppm (m/m) is feasible.

Using the broader bandpass filter for TDI in MCB, a limit of detection (LOD) below 100 ppm (m/m) was estimated with an average time of 1 min [22]. A closer look at these data shows that a 100 ppm (m/m) solution yields an absorbance change ΔA of 3.7 mAU with a standard deviation of σ ≈ 1.7 mAU. In this case, an NEC (1 σ) ≈ 46 ppm (m/m) indicated that a limit of detection below 100 ppm (m/m) is feasible for TDI. However, longer averaging was not feasible with this setup because temperature changes induced signal drifts, and stable operation over more than 10 min was hardly possible. Further details can be found in [22].

### 2.2. Results on Isocyanate Solutions Using a Planar ATR Sensor with a FPI Microspectrometer

**Figure 2:**
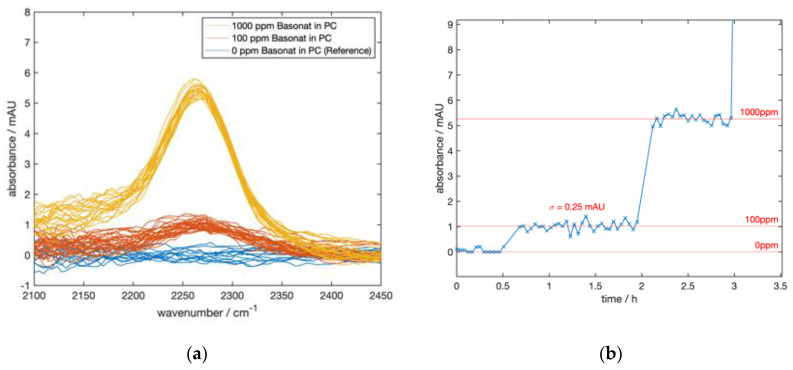
(**a**): FPI spectra of Basonat solutions in propylene carbonate (PC) using the planar ATR setup. Each spectrum was recorded within 150 s and smoothed with a moving average filter. (**b**) Concentration steps derived from (**a**) by a linear fit procedure based on an averaged 10,000 ppm (m/m) spectrum. The total measurement duration was around 5 h (including a 10,000 ppm (m/m) solution not shown here).

Later, drift was effectively reduced by a more rigid mechanical design and compensation by additional temperature sensors. The sapphire crystal has the same dimensions and number of reflections. The setup now uses an FPI microspectrometer (InfraTec LFP-3850C-337) and records a spectrum with 121 spectral data points between 2000 and 2632 cm^−1^ in approximately 150 s with the chosen parameters. The performance was evaluated with Basonat solutions in a variety of concentrations between 0 and 10,000 ppm (m/m) in propylene carbonate. Figure 2 shows the absorbance spectra of 0 (m/m), 100 (m/m), and 1000 ppm (m/m) solutions, calculated from single-channel spectra with pure propylene carbonate as the reference. Each spectrum was smoothed with a 10-point moving average filter to clearly reveal the amplitude of the Basonat absorption around 2260 cm^−1^ and baseline corrected for clarity. In order to evaluate a quantification from those data, we performed a linear fit based on an average 10,000 ppm (m/m) spectrum on each of those spectra. Figure 2b shows the resulting peak heights plotted against time. The shown excerpt demonstrates distinct absorbance changes when switching from 0 to 100 ppm (m/m), and gives a noise equivalent concentration estimate (NEC (1 σ)) of around 25 ppm (m/m) Basonat for the 150-s-spectra (calculation based on the 100 ppm (m/m) measurements (NECs for the other concentrations vary slightly due to non-linearity). The test series was performed over ca. 5 h showing no critical drift or deteriorations, demonstrating the improved stability of the setup.

### 2.3. FTIR Reference Spectra with a Planar ATR Element

For the initial tests of new ATR setups, non-toxic and easy to handle materials with similar spectral features as isocyanate solutions are advantageous. Pressurized CO_2_ could be used at first hand; however, its spectral features are slightly off the isocyanate absorption peak. Compared to liquid samples, it can be rapidly exchanged to non-absorbing N_2_, and fast pressure changes are easy to achieve. However, the refractive index is similar to air and much different from the solvents we used for the isocyanate samples. Acetonitrile is a better alternative. It is not very toxic and mixing with propylene carbonate and isopropanol is possible.

**Figure 3:**
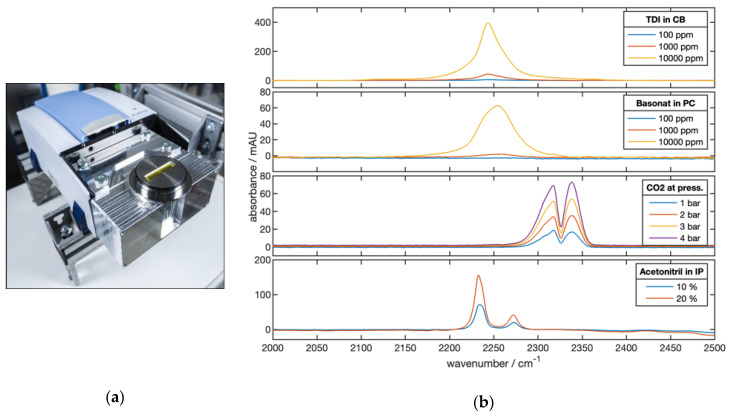
(**a**) Planar ATR element with Varivent flange coupled to a Bruker Alpha FTIR instrument. The system can be attached to a liquid or gas flow system capable for pressure levels of up 5 bar. (**b**) Measured ATR spectra of TDI in MCB, Basonat in propylene carbonate (PC), gaseous CO_2_, and acetonitrile in isopropanol (IP).

To obtain comparable ATR spectra over a broad spectral range and good resolution, a Varivent flange of a process device was equipped with a ZnSe crystal and coupled to an Alpha FTIR spectrometer (Bruker Optik GmbH, Ettlingen, Germany) (Figure 3a). With this setup, 11 ATR reflections were achieved. The spectral resolution was 2 cm^−1^. From the spectra displayed in Figure 3b, the relations shown in Table 2 were obtained. Obviously, TDI in MCB has the strongest absorption compared to the other materials. For a 1-min integration time (i.e., 48 spectral scans), a standard deviation of σ ≈ 0.3 mAU was determined.

## 3. Cylindrical Sensor Concept and Experimental Realization

### 3.1. Concept of a Future Process Instrument with a Cylindrical ATR Element

**Figure 4:**
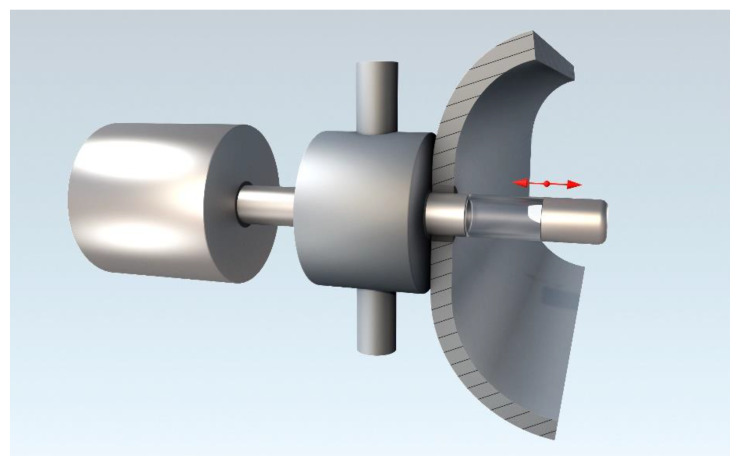
Concept view of a future process probe with a cylindrical ATR element. The probe consists of a tubular shaft with an integrated tubular sapphire ATR element. On one end of the shaft is the sensor control unit with signal processing and driver electronics, on the other end is a sealing cap. Between the process chamber and the control unit the shaft passes through a cleaning chamber.

As described in the introduction, an ATR probe for industrial processes has to have smooth and easy to clean surfaces. A seamless cylindrical surface would be favorable. Automatic liquid and vapor cleaning can be assisted by an adapted tube wiper ring. However, how to integrate such an element into a robust process probe? The basic idea of our project is to integrate a sapphire tube into a stainless-steel probe with negligible surface modification at the interface of the two materials. This probe shaft can be smoothly moved back and forth through suitable O-ring seals as shown in Figure 4. This movement can be considered as a first mechanical cleaning step. In a measurement position, the sapphire tube is exposed to the process medium. In a retracted position, the end cap of the shaft is sealing the port. In this position, the ATR element may be automatically cleaned and/or a recalibration may be performed in a cleaning chamber. In- and out-coupling of infrared radiation into the sapphire element is done from the inside of the tube. Additionally, a temperature sensor may be integrated into the sealing cap to measure the process temperature.

### 3.2. Optical Simulation of Tubular ATR Configurations

**Figure 5:**
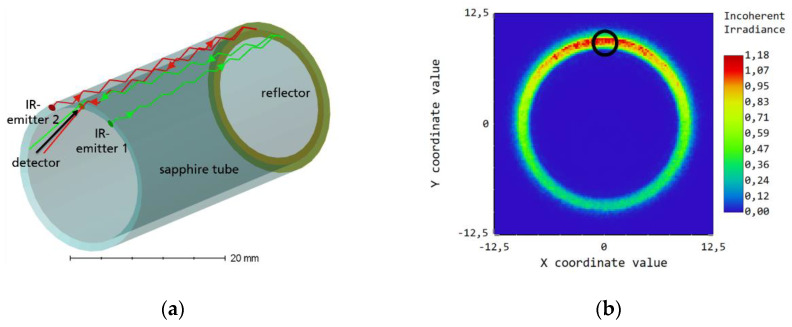
(**a**) Raytracing concept for a tubular ATR element with two infrared emitters placed at a wedged entrance side and a gold-coated reflector at the other side. (**b**): Simulated intensity distribution of the IR radiation, which is reflected from the mirror (direct reflections of the radiation from the first sapphire surface are not shown) on the entrance side. The black ring indicates the position of the IR detector. The false color scale on the right side describes in a. u. the irradiance on the exit area of the sapphire tube. The total exiting intensity is ≈ 26% of the entering intensity of the two emitters.

To investigate the feasibility of such a system, we focus here of the use of tubular ATR elements. Based on the experience with planar ATR sensors employing small thermal emitters and detectors (Section 2), it is obvious that sufficient infrared intensity at the detector entrance and enough suitable internal reflections at the outer surface of the ATR element are the key challenges of such a setup. Minimization of stray light influence is a further objective. Thus, extensive optical simulations using the ray tracing software Optic Studio 19 (Zemax Europe, Ltd., Stansted, UK) were performed to compare ATR elements of a tubular shape or of tube segments with those of a planar geometry and to optimize the positioning of the emitters and detector. The result is shown in Figure 5 for a sapphire tube with a wedged entrance side and an Au-coated reflector at the other side. Two infrared emitters are placed at the entrance and the detector is placed between them. The simulations show that only ≈ 1.8% of the radiation intensity of the two emitters entering the sapphire tube is detected by a LRM-254 detector placed between the two infrared emitters.

### 3.3. Experimental Details

**Figure 6:**
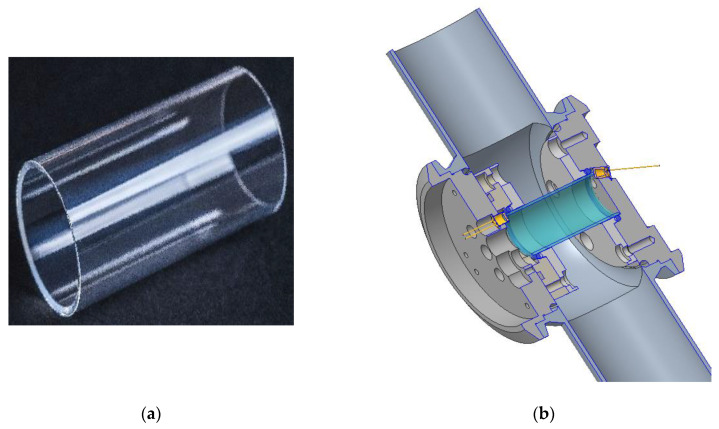
(**a**) Sapphire tube with the 69° wedge on one side. (**b**) Schematic drawing of the mounted ATR element in a flow tube setup with one inclined IR emitter at the right side and one IR detector at the left side of the tube for the ATR transmission experiment.

Based on the optical simulations described above, cylindrical sapphire tubes of 40 mm length, 20 mm outer diameter, and 1 mm wall thickness were obtained from IMPEX HighTech GmbH, Rheine, Germany and a 69° wedge was manufactured by grinding and optical polishing on one end of the tubes (Figure 6a). Some of the tubes were coated with a reflective Au layer on the other side of the tubes. By 90° cutting, polishing, and subsequent Au coating of the sidewalls, a couple of segments of the sapphire tubes were prepared.

The sapphire tube was mounted between two Varivent flanges using O-ring seals (Figure 6b). This module can be mounted in a process flow cell using an outer pair of O-rings and can be pressurized up to 5 bar. For the ATR transmission experiments, three infrared emitters (JSIR350-5-BL-R-D3.6-2-A4, Micro-Hybrid Electronic GmbH, Hermsdorf, Germany) were positioned at the 69° wedge, with the emitter in the middle located just opposite the infrared detector, with integrated filters (Table 1) on the other side of the tube. For the ATR reflection experiments, two emitters were used, and the infrared detector was positioned between the emitters close to the wedged rim of the sapphire tube.

## 4. Experimental Results with Cylindrical ATR Elements

### 4.1. ATR Setup in Transmission Geometry

#### 4.1.1. CO_2_ Pressure Steps

**Figure 7:**
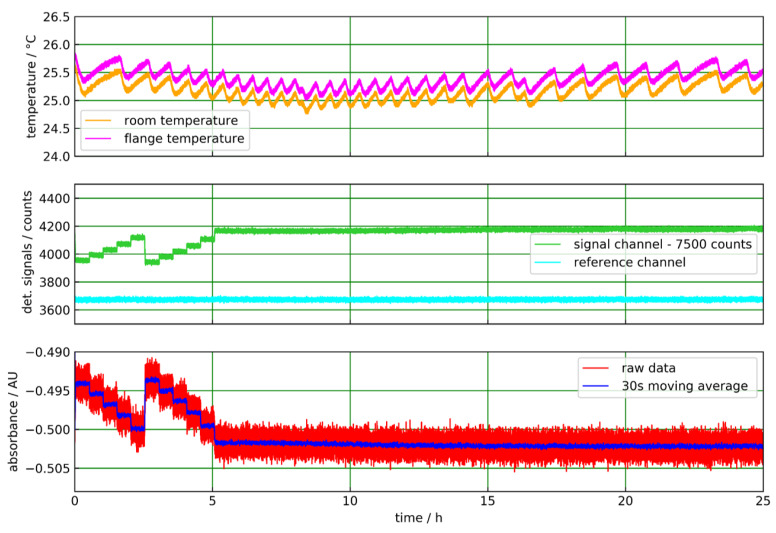
ATR measurement of pure CO_2_ gas with the sapphire tube in transmission geometry (Figure 6b). Ambient and flange temperatures show a sawtooth-like modulation between 25 and 26 °C, which is caused by the lab temperature control. (**top**). The detector signal channel (**middle**) shows characteristic steps when the CO_2_ pressure is varied in 1-bar steps from 5 bar absolute pressure to ambient pressure. After 5 h, a purge flow of N_2_ at ambient pressure sets in. The reference channel is not affected by the pressure variations and stays almost constant. In the absorbance plot the pressure steps are well resolved (**bottom**).

To obtain a first impression of the sensor performance and check for leaks, the flow tube of Figure 6b was pressurized with pure CO_2_ gas. The sealed inner part of the sapphire tube was previously filled with dry N_2_. The CO_2_ pressure can be rapidly changed and was varied in steps from 5 bar absolute pressure to ambient pressure (Figure 7). For this experiment, an LRM-202 detector with a suitable filter (Table 1) was employed, which fits well to the CO_2_ absorption band (Figure 3b). In the absorbance plot (Figure 7 bottom), the pressure steps are well resolved, and the absorbance value for a purge flow of N_2_ at ambient pressure is constant for 20 h. Due to the good stability of the measurement, noise is efficiently reduced by a 1-min moving average.

#### 4.1.2. Acetonitrile in Isopropanol Solutions

**Figure 8:**
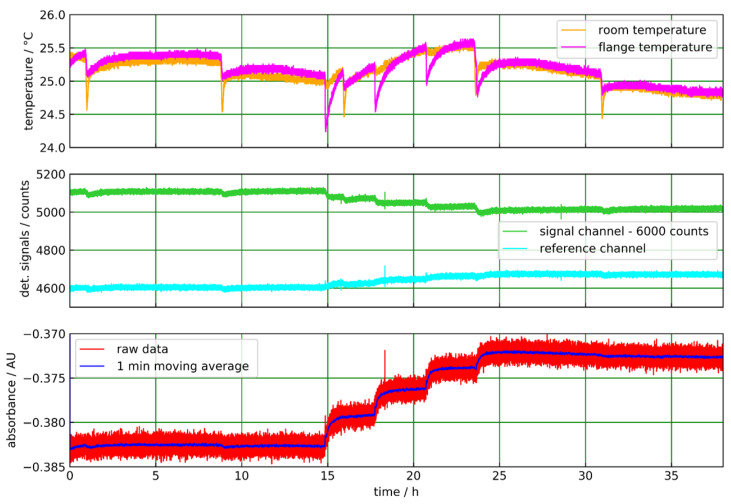
ATR measurement of acetonitrile in isopropanol with the sapphire tube in transmission geometry. The ambient and flange temperatures show distinct dips, which occurred when liquid was added to the sample reservoir (**top**). The signal and reference channel data are slightly affected by these events. Concentration steps (0%, 5%, 10%, 15%, 20% (m/m) acetonitrile in isopropanol) are clearly visible in the signal channel but also in the reference channel, which shows an increasing level at higher acetonitrile concentrations (**middle**). In the absorbance plot the concentration steps are clearly resolved (**bottom**).

The results of the continuous measurement of solutions of acetonitrile in isopropanol for more than 30 h is shown in Figure 8. The solutions are continuously pumped through the abovementioned flow cell with a peristaltic pump at a constant flow rate of approximately 200 mL/min in a closed loop. For the concentration steps, defined amounts of acetonitrile are added to the isopropanol solvent into a liquid reservoir within the liquid flow cycle. The temperature of the liquid reservoir stabilized between 20 and 26 °C. One sensor measures the temperature of the Varivent flange. Another sensor measures the lab temperature in the vicinity of the flange. When adding liquid to the reservoir, the temperature of the flange is changed, and the room temperature value is also affected (Figure 8 (top)).

In this experiment, we employed an infrared detector equipped with a filter, which fits better to the acetonitrile absorption peak in Figure 3b (Table 1). In the absorbance plot (Figure 8 bottom), the concentration steps are well resolved, and the absorbance value for the pure solvent is almost constant for the initial 15 h. Due to this good stability, noise is efficiently reduced by a 1-min moving average. A slight nonlinear response is found in the absorbance steps, i.e., the absorbance changes for relative 5% (m/m) steps of the acetonitrile concentration decrease with an increasing concentration. Such behavior was also observed for the ATR measurements of Basonat and TDI in [22].

### 4.2. ATR Setup in Reflection Geometry

#### 4.2.1. CO_2_ Pressure Steps

**Figure 9:**
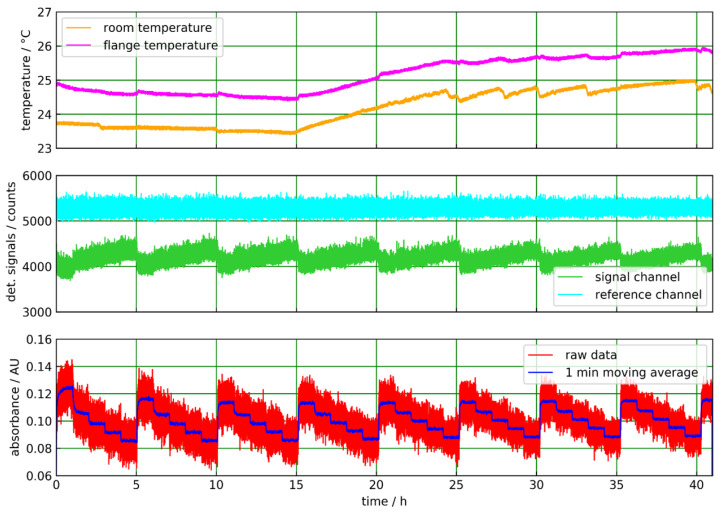
ATR measurement of pure CO_2_ gas with the sapphire tube in reflection geometry. Ambient and flange temperatures are shown in the (**top**) part. The detector signal channel shows characteristic steps when the CO_2_ pressure is varied in 1-bar steps from 5 bar absolute to ambient pressure. The reference channel is not affected by the pressure variations and stays constant (**middle**). In the absorbance plot the pressure steps are clearly resolved by a 1-min moving average (**bottom**).

With the reflection setup, a CO_2_ experiment was also performed first and was pressurized with pure CO_2_ gas. The sealed inner part of the sapphire tube was previously filled with dry N_2_. The CO_2_ pressure was varied in steps from 5 bar absolute to ambient pressure (Figure 9) several times for more than 40 h. For this experiment, the same detector as for the corresponding transmission experiment was employed (Table 1). In contrast to the transmission geometry, the detected power in the signal channel is lower than in the reference channel. The reason is that the optical path is longer for the reflection setup and the infrared absorption of sapphire is higher at the signal wavelength. The signal and reference channel raw data are much noisier than the transmission data. This is caused by the lower infrared intensity at the detector for the reflection setup compared with the transmission geometry. However, in the absorbance plot (Figure 9 bottom), the pressure steps can be well resolved when a 1-min moving average is employed. Due to the good stability of the measurement, even longer integration times may be applied depending on the application.

#### 4.2.2. Acetonitril in Isopropanol Solutions

**Figure 10:**
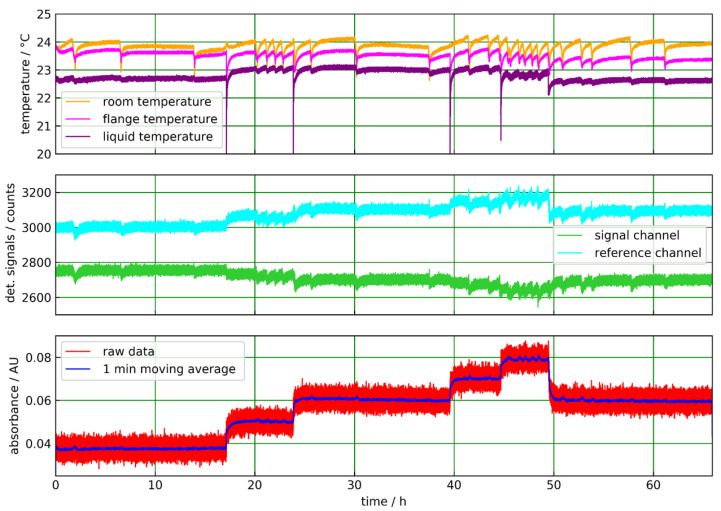
ATR measurement of acetonitrile in isopropanol with the sapphire tube in reflection geometry. Ambient and flange temperatures show distinct dips, which occurred when some liquid was added to the sample reservoir (**top**). The signal and reference channel data are hardly affected by these events. The concentration steps (0%, 5%, 10%, 15%, 20% (m/m) acetonitrile in isopropanol) are clearly visible in the signal channel but also in the reference channel, which shows an increasing level at higher acetonitrile concentrations (**middle**). In the absorbance plot the concentration steps are clearly resolved. Noise is efficiently reduced by a 1-min moving average (**bottom**).

The experiment was performed in a similar way as with the transmission setup, again using the LRM-254 detector with the previous filter combination (Table 1). In the absorbance plot (Figure 10 bottom), the concentration steps are well resolved, especially when averaging over 1 min, and the absorbance value for pure solvent is almost constant for the initial 17 h. Additionally, the value for 10% (m/m) acetonitrile is almost constant and reproducible for two time intervals of more than 15 h. The mentioned nonlinearity is smaller than in the transmission setup.

## 5. Discussion

We showed that ATR measurements on liquids and gases can be performed using tubular ATR elements and compact thermal sources and infrared detectors. Here, we analyzed and compared the signals and noise features of the different setups in more detail. For this purpose, we calculated the Allan deviations of the baseline signals of the CO_2_ and acetonitrile measurements of Section 4. The Allan deviation was first introduced by Allan et al. to describe the statistical uncertainty of atomic frequency standards [23]. When taking a series of measurements of, e.g., the frequency of a clock, the Allan deviation σ_A_ of the frequency first decreases with increasing integration time τ (or number of averaged consecutive observations) because the noise averages out. For long integration times, the Allan deviation increases again due to various drift factors. This concept was later applied by Werle et al. [24] to describe the stability of laser spectrometers and now is generally used to describe the performance of various measurement systems.

**Figure 11:**
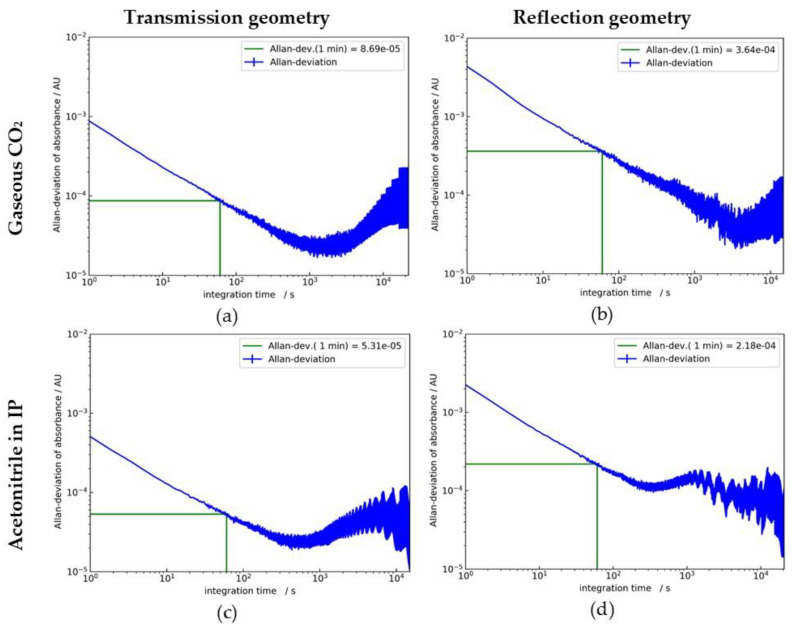
Allan deviation plots for ATR measurements with sapphire tubes. (**a**): CO_2_ pressure steps transmission setup; (**b**): CO_2_ pressure steps reflection setup; (**c**): acetonitrile in the isopropanol transmission setup; (**d**): acetonitrile in the isopropanol reflection setup. Allan deviations for an averaging time of 1 min were determined from the plots as indicated.

All plots in Figure 11 show a decrease of the Allan deviation σ_A_ proportional to τ^−1/2^ over more than 2 decades of the integration time τ. This is typical for white noise. The Allan minimum, indicating the averaging time τ_min_ for the lowest σ_A_ value, is reached at roughly one hour for the CO_2_ measurements and between 5 and 10 min for the liquid measurements. For longer integration times, σ_A_ is increasing due to drift. However, even at the longest integration times, the corresponding σ_A_ is not considerably exceeding the values for an integration time τ of 1 min, which are used in Figure 7, Figure 8, Figure 9 and Figure 10, in [22], and listed in Table 3. Depending on the application, longer integration times of several minutes are an option for further noise reduction. Overall, the data demonstrate a high stability of the measurements.

If we compare σ_A_ (1 min) for CO_2_ and acetonitrile, the values for the liquid measurements are somewhat lower than for CO_2_, probably due to the different indices of refraction and detector types. Comparing transmission and reflection, it is obvious that σ_A_ for the transmission measurements is lower by a factor of ≈ 4.1 because of the lower infrared radiation intensity at the detector for the reflection setup.

Table 3 shows the absorbance changes for the 1% (m/m) acetonitrile solution respectively 1-bar CO_2_ pressure steps for the transmission and reflection setups obtained from Figure 7, Figure 8, Figure 9 and Figure 10. The absorbance changes for the reflection setup are a factor of ≈ 3.7 higher than for the transmission setup, but this is compensated by lower Allan deviations. As a result, the NEC (1 σ) values for reflection are only approximately 10% higher than for transmission. This indicates that the effective number of reflections is almost twice the transmission value, despite the strong divergence and scattering effects in the tubular geometry. The NEC (1 σ) figures suggest, that acetonitrile concentrations of 1000 ppm (m/m) or CO_2_ pressure changes of ≈ 50 mbar could be detected with both setups. Doubling these values is a more realistic estimate for the achievable limits of detection (LOD).

The NEC (1 σ) values for the planar photometer setups in Table 4 are quite similar to the data for the cylindrical geometries, keeping in mind that conversions and digitization may introduce some uncertainties. The FPI measurement gave a substantially lower NEC (1 σ) value for Basonat. This is partly due to the longer averaging time but may also result from the more advanced spectral evaluation. Future experiments with the cylindrical ATR setups and FPI detectors may clarify this point.

## 6. Conclusions and Outlook

Compared to common ATR probes, which are using sapphire or diamond prism [6,14,15,16] tips with 2-3 reflections or a diamond plate as a chemically resistant ATR window attached to a ZnSe crystal (1 reflection) [25], planar ATR elements have a higher sensitivity due to more reflections. With this setup, an isocyanate concentration below 100 ppm (m/m) can be determined [22]. In this work we showed that sensitive IR-ATR sensors can be realized using sapphire tubes as cylindrical ATR elements. With such elements, having a comparable length to usual planar ATR crystals, similar detection limits can be achieved for transmission and reflection setups. Isocyanate solutions with concentrations below 100 ppm (m/m) can be detected with a photometer device. Cylindrical ATR sensors have a smooth and easy to clean surface, will be suitable for CIP procedures, and can be incorporated into a process flow similar to Figure 6, e.g., in an online bypass configuration.

In an alternative configuration to Figure 6, the process medium may flow inside of the sapphire tube. Although this would require several changes of the mechanical construction, similar results are expected when the inner side of the tube serves as the active ATR surface. Easy cleaning of the inner side of a sapphire tube is also possible.

Furthermore, such sapphire tubes could be incorporated into a process probe with an automatic cleaning system as shown schematically in Figure 4. However, for this purpose, a seamless integration of the sapphire tube into a stainless-steel shaft and an efficient coupling of the infrared emitter and detector elements have to be realized. For most industrial applications, such a probe has to comply with safety regulations (e.g., explosion prevention) and has to withstand temperatures up to 200 °C. Thus, infrared emitters and the detector may not be attached directly to the sapphire tube and additional light guides will be required. Coupling of sufficient infrared radiation into the sapphire tube is even more challenging for a process probe.

First, experiments with sapphire segments (Section 4) indicated that compared to the tubes, similar results were obtained with less incident power, and a higher radiation throughput can be achieved. This may facilitate coupling at the expense of a more complex mechanical integration.

All limitations by low radiation intensities will be overcome by novel MIR laser sources. They offer a huge intensity increase compared to thermal light sources, which are fundamentally limited by Planck’s law. Recently, real-time reaction monitoring using ATR spectroscopy with a dual comb spectrometer was demonstrated [26]. Although still a rather expensive and bulky approach, the combination of rugged cylindrical ATR probes and such light sources will enable new process analytical applications.

## Figures and Tables

**Table 1:** IR filters and detectors used for planar and cylindrical photometer configurations. The filters were supplied and integrated by InfraTec GmbH into the detector housings. Data are typical datasheet values from InfraTec GmbH.

Filter-No.:	Planar (P) or Cylindrical (C) Use	Center Wavenumber (CWL) /cm^−1^	Half Power Bandwidth (HPBW) /cm^−1^	Peak Transmittance /%	Detector Type (Element Size)
1	P-signal#1 [22]	2350	100	81	LMM-242 (2 × 2 mm^2^)
2	P-signal#2 [22]	2345	330	90	LMM-242
3	P-reference#1&#2 [20]	2530	60	88	LMM-242
4	C-signal CO_2_	2342	93	88	LRM-202 (1.2 × 0.8 mm^2^)
5	C-reference CO_2_	2532	58	83	LRM-202
6	C-signal acetonitrile	2257	138	90	LRM-254 (1.4 × 1.4 mm^2^)
7	C-reference acetonitrile	2532	58	84	LRM-254

**Table 2:** Peak absorbances of sample spectra from Figure 3b. Values for the second maxima are in brackets. The relative absorption strength is calculated for the main peaks.

Sample	Spectral Position of Maximum Absorbance/cm^−1^	Full Width at Half Maximum (FWHM)/cm^−1^	Absorbance at Spectral Position of Maximum Absorbance/mAU	Maximum Absorbance for 1% (m/m) Solution Resp. 1 Bar Pressure of CO_2_/mAU	Relative Absorption Strength Compared to Acetonitrile in Isopropanol
**Acetonitrile in isopropanol**	2230 (2270)	20 (13)	155 (41) for 20% (m/m)	7.75	1.0
**TDI in MCB**	2240	50	395 for 1% (m/m)	395	51.0
**Basonat in propylene carbonate**	2255	80	63 for 1% (m/m)	63	8.1
**Gaseous CO_2_**	2338 (2317)	20 (30)	73 (69) for 4 bar	18.25	2.35

**Table 3:** Analysis of the ATR experiments with sapphire tubes. NEC (1 σ)-values for Basonat respectively TDI are estimated using the conversion factors from Table 2.

Sample	Absorbance Change for 1% (m/m) Solution Resp. 1 Bar CO_2_ Pressure Step/mAU)	Allan Deviation σ_A_ (1 min)/mAU	NEC (1 σ)/ppm (m/m) resp. mbar	Estimated NEC for Basonat/TDI/ ppm (m/m)
	**Transmission**			
**Acetonitrile in isopropanol**	0.68	0.053	780	96/15
**Gaseous CO_2_**	1.85	0.087	47 mbar	136/22
	**Reflection**			
**Acetonitrile in isopropanol**	2.6	0.22	846	104/17
**Gaseous CO_2_**	6.63	0.36	54 mbar	157/25

**Table 4:** Data of ATR transmission experiments with sapphire plates.

Sample	Absorbance Change/mAU	Standard Deviation (Averaging Time τ)/mAU	NEC( 1 σ)/ppm (m/m)
**Basonat in propylene carbonate (photometer)** Digitized data from [22]	7.05 (4600 ppm (m/m))	0.14 (1 min)	91
**TDI in MCB (photometer)** Digitized data from [22]	3.7 (100 ppm (m/m))	1.7 (1 min)	46
**Basonat in propylene carbonate (FPI)** (s. chapter 2)	1 (100 ppm (m/m))	0.25 (2.5 min)	25
